# Incorporating rich background knowledge for gene named entity classification and recognition

**DOI:** 10.1186/1471-2105-10-223

**Published:** 2009-07-17

**Authors:** Yanpeng Li, Hongfei Lin, Zhihao Yang

**Affiliations:** 1 Department of Computer Science and Engineering, Dalian University of Technology, Dalian, PR China

## Abstract

**Background:**

Gene named entity classification and recognition are crucial preliminary steps of text mining in biomedical literature. Machine learning based methods have been used in this area with great success. In most state-of-the-art systems, elaborately designed lexical features, such as words, n-grams, and morphology patterns, have played a central part. However, this type of feature tends to cause extreme sparseness in feature space. As a result, out-of-vocabulary (OOV) terms in the training data are not modeled well due to lack of information.

**Results:**

We propose a general framework for gene named entity representation, called feature coupling generalization (FCG). The basic idea is to generate higher level features using term frequency and co-occurrence information of highly indicative features in huge amount of unlabeled data. We examine its performance in a named entity classification task, which is designed to remove non-gene entries in a large dictionary derived from online resources. The results show that new features generated by FCG outperform lexical features by 5.97 F-score and 10.85 for OOV terms. Also in this framework each extension yields significant improvements and the sparse lexical features can be transformed into both a lower dimensional and more informative representation. A forward maximum match method based on the refined dictionary produces an F-score of 86.2 on BioCreative 2 GM test set. Then we combined the dictionary with a conditional random field (CRF) based gene mention tagger, achieving an F-score of 89.05, which improves the performance of the CRF-based tagger by 4.46 with little impact on the efficiency of the recognition system. A demo of the NER system is available at .

## Background

With the explosion in volume of biomedical literature, developing automatic text mining tools has become an increasing demand [1]. Extracting biological entities, such as gene or protein mentions, from text is a crucial preliminary step, which has attracted a large amount of research interests in the past few years. The results of some challenge evaluations, such as BioCreative [2], BioCreative 2 [3] and JNLPBA [4], show that significant progress has been made in the tasks of bio-entity tagging. BioCreative 2 gene mention (GM) task [3] is the most recent challenge for gene named entity recognition, where a highest F-score of 87.21 is achieved [5].

In these tasks, machine learning based methods have shown great success, which are used by all the top-performing systems. Feature representation is an important issue. Apparently, the state-of-the-art systems in these challenges [5-10] used very similar feature sets, such as words, n-grams, substrings, morphology patterns (including word shapes, domain-specific regular expressions, etc.) and part-of-speech (POS) tags.

It is well known that the lexical-level features have played a central part in named entity recognition/classification (NER/C) tasks. However, this type of feature tends to cause extreme sparseness in feature space, where out-of-vocabulary (OOV) terms are not modeled well. In the task of gene NER/C, the impact of OOV terms is large, since there are millions of gene/protein names mentioned in biological texts and new names are created and described all the time.

The introduction of substring and morphology features has made the OOV term recognition feasible, because many gene names are generated under certain morphology rules. However, there are a large number of gene names that 'look like' common English words or entities of other types, where these features may not work well. In all, the main drawback of lexical level representation is the lack of background knowledge for a large number of OOV terms and 'surface insensitive' terms. As is argued by Lanczos [11], 'a lack of information cannot be remedied by any mathematical trickery'. One interesting question is: is there any 'higher-level' feature set that can lead to similar or better performance?

There are many approaches applied to overcome the data sparseness by incorporating external knowledge, including features derived from POS tags, external gazetteers [5,7-9], Web search [8], and unlabeled MEDLINE records [5]. According to their reports, these additional features lead to an increase in performance of up to 2%. We wonder if these methods can utilize the background knowledge well and if there is space for further improvement.

POS tags are not strong indicators for NE identification, and always need to be combined with other features. Finkel *et al*. [8] showed that given large amount of training data (the BioCreative training corpus), the improvement of POS tags over lexical features became very slight.

With the increasing amount of genomics databases, e.g., Entrez Gene or UniProt, dictionaries derived from these resources are expected to enhance the NER system. Dictionary look-up is the most straightforward technique for NER, but its performance for gene entity recognition is inferior to machine learning methods [2-4]. In another way, many researchers use dictionary match results as features of linear discriminative classifiers [5,7-9]. However, as reported in their works, the improvements are consistent but not large. It seems that these rich resources are not utilized well. We attribute the main reasons to the noisy terms and low coverage of the dictionaries, which will be discussed further in the following sections.

Ando [5] used alternating structural optimization (ASO) semi-supervised learning method [12], which generates new features through linear combination of original features by optimizing on a large number of auxiliary problems in unlabeled data (a subset of MEDLINE). This method achieved an improvement of 2.09 F-score over a baseline of 83.9 on BioCreative 2 test data [5].

Finkel *et al*. [8] employed a Web-based method to filter false positive gene candidates using features derived from the co-occurrence of the current name and a strong indicative context (including '*gene'*, '*mutation' *and '*antagonist'*) on the Web. The contribution of this feature is 0.17 F-score according to their result analysis.

A similar method can be found in entity classification for general domain. Etzioni *et al*. [13] developed an information extraction system called *KnowItAll*, which bootstraps semantic dictionaries from the Web. In the procedure of dictionary refinement, they used pointwise mutual information (PMI) of a name candidate and 'discriminator phrases' (such as '*X is a city*') to assess the likelihood that the candidate belongs to the class. The PMI scores were obtained from the Web search engine and were treated as features of a naïve Bayes classifier.

The Web-based methods [8,13] and ASO are all able to generate new features from unlabeled data. The primary advantage of the Web-based methods over ASO and other semi-supervised learning methods surveyed in [14] is that they are simple to implement and don't need complex matrix computation or function optimization so that huge amount of unlabeled data can be utilized easily. However, they are not systematically studied compared with elaborately designed lexical-level features and are not general enough to be extended to a broader area of text mining or machine learning.

Addressing these problems, we propose a general framework for creating higher-level features using the relatedness of highly indicative features in huge amount of unlabeled data, which gives a general interpretation of the Web-based methods[8,13]. We examine its performance in a gene named entity classification (NEC) task, which is designed to remove non-gene entries for a large dictionary derived from online databases. The performance of this method is compared with elaborately designed lexical features and the impact of each extension based on it is investigated.

**Figure 1:**
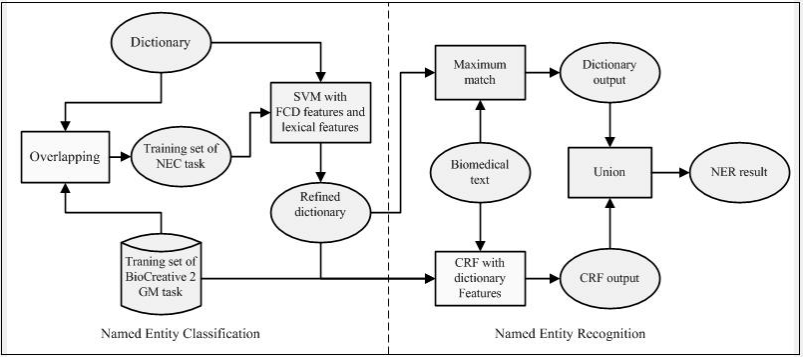
**Framework of the named entity recognition system**.

We also examine the contribution of the refined dictionary in the BioCreative 2 GM task. The reasons are: 1) we are going to show that an automatically generated dictionary is able to achieve state-of-the-art performance in the NER task. 2) The performance can be compared directly with other systems in this challenge. We investigate two approaches for NER: a simple dictionary look-up method and an ensemble method that combines the dictionary and a conditional random field (CRF) [15] model with lexical features. Therefore our final system consists of two stages. The first is to learn a high quality dictionary in an offline manner. The second is to use the dictionary (possibly combined with other methods) to locate gene names in texts. The system architecture is shown in Figure 1.

## Feature coupling generalization

In this section, we describe our framework for feature generation from unlabeled data. We call this approach feature coupling generalization (FCG). The basic idea is to construct new features from the relatedness measures of two types of 'prior features'. In the following sub-sections, we discuss how to obtain the relatedness measures and how to convert them into new features.

### Relatedness of indicative features

We first investigate the factors we may consider in order to generate a good binary feature for NER/C tasks. Intuitively, when selecting a feature, two factors should be considered, i.e., the distinguishing abilities for classes and for examples. If neither of them can be guaranteed, the feature will inevitably be irrelevant. These two factors lead to two strategies for feature generation. In one case, if we are familiar with the target problem, we will directly choose the features with highly distinguishing ability for the classes. For example, when determining whether a term is a gene name, we will choose patterns ending with '*gene*' or '*protein*' as features. In another case, we are not confident what good features are, but intuitively a feature that has the strong capability to differentiate the current example from others tends to be a good candidate. For instance, in NER or NEC tasks, word or n-gram features can be attributed to this class.

We define the first type of features as class-distinguishing features (CDFs) and the second type as example-distinguishing features (EDFs). It is not difficult to understand that the co-occurrence of highly indicative EDF and CDF can be a strong indicator for classification, whether it appears in labeled or unlabeled data. In particular, these co-occurrences can give additional valuable information for classification when limited training data is labeled while huge amount of unlabeled data is available, since it is possible to get much more indicative feature co-occurrences from unlabeled data.

Next we will give a framework on how to generate new features using the feature co-occurrence information. To clarify the idea, we first give the concept of feature coupling degree (FCD): given an EDF *f*_*e*_, a CDF *f*_*c*_, and an unlabeled data set *U*, the FCD measures the relatedness degree of *f*_*e *_and *f*_*c *_in *U*, denoted by *FCD *(*U*, *f*_*e*_, *f*_*c*_). Note that we do not use a specific formula here, because our goal is to investigate the classification performance using FCDs as features. In this case, different classifiers may prefer different feature types. Also one specific formula is not certain to give the best result, which means that by combining several FCD measures by the target classifier better classification performance can be expected.

### Learning a general representation

In order to convert FCDs into new features, the following issues should be considered: how to index the new features? Which attributes of examples do the FCDs reflect? The basic idea of our solution is to index the new features using the conjunction of higher-level attributes of EDFs (called EDF roots), CDFs and the types of FCD.

In machine learning, features can be viewed as a set of attributes that are used to describe different examples. Usually there are hierarchy relationships between these attributes in a specific problem. We consider a simple case where there are two levels of attributes. Let *H *= {*h*_1_,..., *h*_*m*_} be the higher level attributes and *L *= {*l*_1_,..., *l*_*n *_}be the lower level ones. For any *l *∈ *L *there exists an *h *∈ *H*, such that *h *= *parent*(*l*), where *parent *: *L *→ *H *is a function that finds the higher level concept of *l *in *H*. In the task of gene NEC, for instance, *H *can be the set'

{*lowercase name'*, *'leftmost 1-gram'*, '*rightmost 1-gram'*, '*bag-of-words'*,...}

and *L *is

{'*lowercase name = il 2 gene'*,..., '*leftmost 1-gram = IL'*,..., *'rightmost 1-gram = gene'*,..., '*bag-of-words = IL', 'bag-of-words = 2'*,....}.

**Figure 2:**
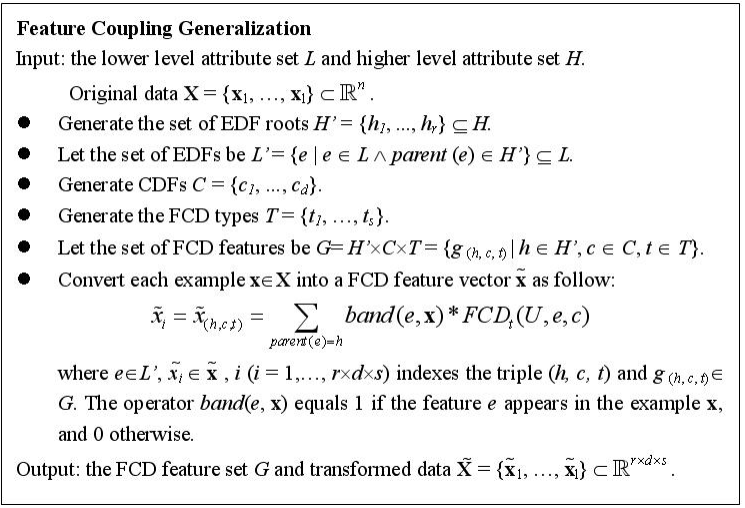
**Feature coupling generalization**.

Note that in this example *n*>>*m*, since each concept in *H *can generate thousands of terms in *L*. In practice, we always choose *L *as the feature set (lexical features) rather than *H*, as the elements in *H *are abstract concepts, for which a numerical or Boolean representation for each example is not obvious. In our framework a subset of *H *is used to index FCD features in conjunction with CDFs and FCD types. Let *C *= {*c*_1_,..., *c*_*d *_}be the set of CDFs and *T *= {*t*_1_,..., *t*_*s *_}be the set of FCD types. The algorithm of FCG is presented in Figure 2.

We call EDFs or CDFs *prior features*, since in the FCG algorithm they are *not final representations *of examples and need to be designated before FCD features. The FCG algorithm can be summarized as follows: 1) Select the two types of prior features and FCD types. 2) Calculate FCDs from the unlabeled data. 3) Construct new features by FCD values and the conjunction of EDF roots, CDFs and FCD types. For example, for classifying the name candidate '*prnp gene*', we can choose EDF, CDF, EDF root and FCD type as {'*leftmost 1-gram = prnp*'}, {'*expression of X*'}, {'*leftmost 1-gram*'} and {*PMI*}. The FCD feature generated by them is indexed by

'*leftmost 1-gram*'∧'*expression of X*'∧'*PMI*'

and its value is

*FCD*(*U*, *'leftmost 1-gram *= *prnp'*, *'expression of X'*) = *PMI*(*'leftmost 1-gram *= *prnp'*, *'expression of X'*) (estimated from *U*).

EDF roots are defined as a subset of *H *which has the relatively strong distinguishing ability for the current example, such as {'*lowercase name'*, *'leftmost 1-gram'*, '*rightmost 1-gram'*} for entity classification. Intuitively, there are two factors that may influence the quality of EDFs: the indicative capability and the information obtained from unlabeled data. On the one hand, if the features are weak indicators for the current example, its co-occurrence with CDFs tends to be weak features. On the other hand, if the features are 'over-indicative' (e.g., extremely low frequency even in the unlabeled data), sufficient information could not be obtained from unlabeled data to train a good classifier. So when selecting EDF roots and EDFs, a trade-off between these two factors should be considered.

CDFs are the features that are most relevant to the classes we are concerned with. Given the labeled data, the CDFs can be selected via a classical feature selection technique (e.g., information gain or Chi-square). This doesn't mean a good CDF amounts to a good feature in supervised learning. The effects of CDFs should be measured only by the quality of the resulting FCD features. In some cases, a CDF is not required to appear in the labeled data necessarily as long as it is indicative to the classes in the view of the whole unlabeled data. In addition, similar to EDF selection, when selecting CDFs one should also consider the two factors (i.e., indicative and informative). In the following sections, we will analyze the impact of various types of EDFs and CDFs on a gene NEC task in detail.

The FCD type is another important component in this framework. As discussed in the previous section, in practice we can design several formulas to model the coupling degree of an EDF-CDF pair from different aspects for a specific task. In the following sections, several different FCD measures will be compared.

Note that in the last stage of the algorithm (Figure 2), we convert an example **x **into a vector of FCD features using the following equation.

(1)

Here each feature  in the new example  maps a triple (*h*, *c*, *t*) in the FCD feature vocabulary *G*. Given the triple and a specific example **x**, the feature value of each  can be computed in equation (1). Note that in this formula we have considered the situation called 'root conflict', where an EDF root *h *maps multiple non-zero EDFs in one example. Here we used the sum of all the FCDs derived from the same EDF root *h *as the feature value of . For instance, if '*bag-of-words*' is selected as EDF root, 'root conflict' may exist, since one gene named entity may contain multiple 'bag-of-words' features. But for the attributes '*lowercase name'*, *'leftmost 1-gram' *or '*rightmost 1-gram'*, apparently this conflict doesn't exist.

**Figure 3:**
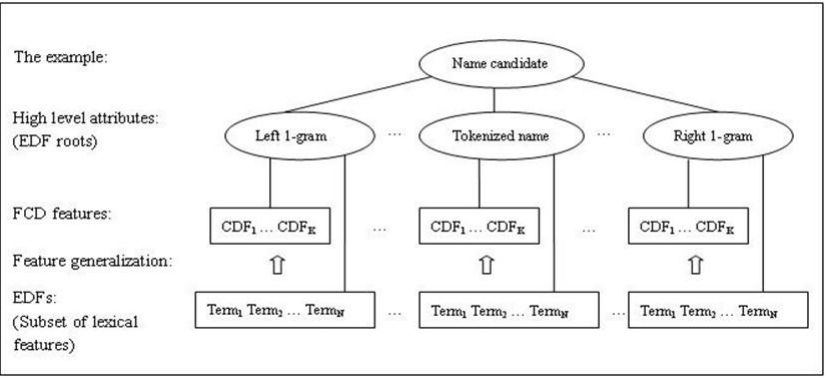
**An example of FCG method applied in the gene named entity classification task**. Here an EDF can be viewed as the conjunction of an EDF root and a term. Only one FCD type is used. A FCD feature is the conjunction of an EDF root and a CDF.

In addition, the FCD features can be assumed as a higher-level (or general) representation of lexical features, which also explains why the classification performance can be enhanced. The FCD features are determined by three components: the EDF roots, CDFs and FCD types. Actually the former two are more general than lexical features. The EDF roots are higher-level concepts of EDFs (a subset of lexical features) as described previously. Since the CDFs reflect some common characteristics of a large number of EDFs, they can be viewed as latent semantic types of EDFs. One could assume that the EDFs are projected to the dimension *H' *× *C *× *T *(Figure 2), which is somewhat related to principle component analysis (PCA). But different from PCA, the 'principle components' in FCD features are closely relevant to the class labels and given in an explicit way. The advantage of the general representation is that it can overcome the data sparseness to a large extent and suffers less from the impact of OOV terms. In Figure 3, an example shows how the features are transformed into higher-levels in an entity classification task.

Obviously the FCG based feature generation method gives a general interpretation of some Web-based methods [8,13], where EDFs are the strings that represent the whole named entity, CDFs are the patterns with indicative contexts, the FCDs are Boolean function [8] and PMI variant [13] respectively. Moreover, they can be greatly extended in our framework, which means that we can exploit various types of EDFs, CDFs, or FCD metrics according to the concrete application. Next we will study the impact of some extensions in a gene named entity classification task.

## Methods

**Figure 4:**
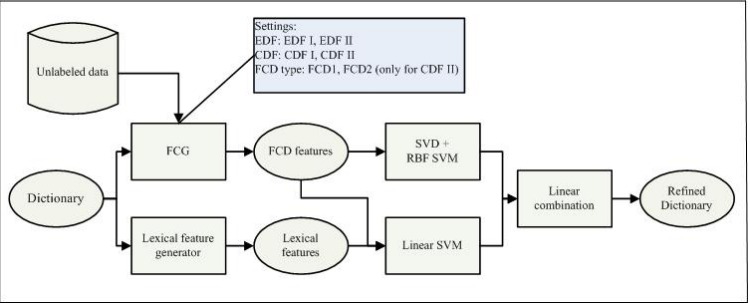
**Components and FCG settings of the final named entity classification system**.

In this section, we detailed each component in the NEC and NER tasks. The NEC task aims to determine whether a dictionary entry is a gene or protein name. We first built a large dictionary using online genomics resources and variant generation. Then we used the overlap of the dictionary and the corpus of BioCreative 2 GM task to generate positive and negative examples for the NEC task, where the performances of lexical features and FCD features were compared. The settings of FCG and data/process flow of our final NEC system are shown in Figure 4. Finally, we examined the contribution of the refined dictionary in the BioCreative 2 GM task. The data/process flow diagram of the whole NER system is shown in Figure 1.

### Dataset for named entity classification

The dictionary was built from two resources: BioThesaurus 2.0 [16] and ABGene lexicon [17]. To improve the dictionary coverage, we converted all the dictionary entries to lowercases, removed hyphens and tokenized the terms from non-letter and non-digit tokens, e.g., *(IL)-7 gene *-> *( il ) 7 gene*. Then variants were generated by removing the boundary 1 and 2 grams of each dictionary entry respectively and adding the new terms to the dictionary. Finally, we got a dictionary containing over 10 millions entries.

Table 1 summarizes the statistical information of these dictionaries. The dictionary coverage is defined as follow:

**Table 1:** Statistical information and NER performance of dictionaries

Dictionary	#of entries	Coverage	Precision	Recall	F-score
BioThesaurus	4,480,469	36.78%	15.36	77.21	25.62
ABGene lexicon	1,101,716	35.98%	31.54	53.58	39.71
Combined	5,522,822	54.32%	16.20	82.59	27.09
Combined+varients	10,034,696	65.68%	7.32	79.26	13.40

The rightmost three columns show the recognition performances of dictionaries on BioCreative 2 test corpus using maximum match method.

(2)

where the numerator is the number of entries both in the dictionary and entity list of BioCreative 2 data including boundary alternatives. In Table 1, it can be seen that the coverage is substantially improved by data combination and variant generation. We also compared the recognition performances of these dictionaries on BioCreative 2 GM task using forward maximum match. As can be seen, the overall performances of these dictionaries are rather poor, especially in precision, revealing that the impact of noise is serious. Note that the recall can not reflect the dictionary coverage actually, because in the BioCreative evaluation script, the false positive predictions at entity boundaries are penalized twice both in precision and recall.

When analyzing the 'noise' in BioThesaurus and ABGene lexicon, we found that there were many common English words (e.g., *similar*), common biological terms (e.g., *binding motif*), language fragments (e.g., *signaling molecules in*), and other entity types (e.g., *DSM-IIIR*, a book). Note that even a dictionary derived from high quality domain-specific databases may not be a good choice for NER, since there are many terms referring to different entities in different contexts. The databases will include a noun phrase as long as it is identified as a gene name in a local context, but the most valuable dictionary entries for the NER task are terms that are recognized as gene names in most cases. Therefore, for a good dictionary-based method, noise caused by all these cases must be removed.

In order to remove noisy terms in the dictionary, we created a named entity classification (NEC) task using BioCreative 2 data set and the dictionary, which is to determine whether a dictionary entry is a gene name in most cases. First, we used a dictionary look-up method to search the plain text corpus of BioCreative 2 GM task, and got two lists of gene name candidates (one from the training and another from the test corpus). The next problem is how to define positive and negative examples, as one term may have different meanings in the different contexts. Here for the ambiguous term we chose the most frequent label in the BioCreative 2 training/test corpus as the gold standard of training/test set of the NEC task respectively. Finally, the NEC training data contains 12567 positive and 36862 negative examples, and test data contains 5297 positive and 18960 negative examples. We used support vector machines (SVMs) as classifiers and compared the performances of lexical features and FCD features.

### Lexical features

Many lexical features can be borrowed from NER, as the two tasks are closely related. In the NEC task, systems don't need to recognize entity boundaries, and entity-level features, such as name lengths, boundary n-grams, are easy to incorporate. After hundreds of experiments, we selected six types of lexical features with a vocabulary size of 444,562. *Bag-of-n-grams *(*n *= 1, 2, 3): the positions of n-grams are ignored in this case.

*Boundary n-grams *(*n *= 1, 2, 3): conjunction of each n-gram at entity boundary and its position (e.g., '*left-2-gram = IL 2*' for '*IL 2 gene*'). We also seek to conjunct all the n-grams (beyond boundary n-grams) with their positions, but the effects were negative, so they were not incorporated into the final runs.

*Sliding windows*: character-level sliding windows with the size of five.

*Boundary substrings*: 1–6 character-level prefix and suffix of the name candidate.

*Morphology features*: we applied morphology patterns widely used in previous NER systems, such as shape (e.g., *his237 *-> *aaa000*), brief shape (e.g., *his237 *-> *a0*), normalized name (e.g., *IL2R alpha *-> *ilDrG*, where '*D*' and '*G*' refer to digit and Greek letter), and name length. In our experiment, only boundary n-grams and the whole names were converted to morphology patterns. We also tried to applied these patterns to all the n-grams and substrings, but found that the performance did not improve while a lot of additional computational cost was introduced. For all lexical features, SVM^light ^[18] with linear kernel was used as the classifier and the trade-off parameter *C *was set at 0.15.

### Generation of EDFs

Considering the characteristics of gene named entity classification and the trade-off between 'indicative' and 'informative' (presented in the prior sections) we selected two types of EDFs:

#### EDF I

tokenized name candidates (e.g., *(P-450scc) gene *-> *( p 450 scc ) gene*). It is nearly the most discriminating features to distinguish the current example from others. In this task, fortunately a large number of co-occurrences of name candidates and CDFs can be obtained from the unlabeled data (detailed in the 'Unlabeled data' sub-section) especially for short terms.

#### EDF II

boundary n-grams. It includes leftmost and rightmost word-level 1-grams, 2-grams and 3-grams at entity boundaries. In this task, it is difficult to incorporate sufficient background knowledge into gene entity representation from the unlabeled data using only EDF I. Since there are a lot of multi-token names and long names of gene entities, many exact co-occurrences of EDF I and CDFs are difficult to get from the unlabeled data. But in this case we can also infer the categories of entities according to the boundary n-grams, since these features tend to be highly indicative on average.

Examples of EDFs are shown in Table 2. The set of all the EDF roots used in our work is:

**Table 2:** Examples of EDFs and CDFs

Feature type	Examples	# of features
EDF I	*Name = human serotonin transporter gene*,	--

EDF II	*Left1 = human*, *Left2 = human serotonin*, *Left3 = human serotonin transporter*, *Right1 = gene*, *Right2 = transporter gene*, *Right3 = serotonin transporter gene*	--

CDF I (left)	*X gene*, *X binding sites*, *X is involved in *...	300
CDF I (right)	*human X*, *kinase ( X*, *the expression of X*...	300
CDF II	*t *> 0.5, *t *> 0.25, *t*> 0, *t*>-0.25, *t *> -0.5, *t *< -0.75, *t *< -1, *t *< -1.25, *t *< -1.5, *t *< -1.75, *t *< -2	11

In the examples of CDF II, *t *is the prediction score given by a SVM trained by local context words.

{*Name*, *Left-1*, *Left-2*, *Left-3*, *Right-1*, *Right-2*, *Right-3*}

where *Name *refers to the tokenized name candidate and *Left-n *or *Right-n *is the leftmost or rightmost boundary n-gram.

### Generation of CDFs

Two general types of CDFs were used in the NEC experiments.

#### CDF I

indicative context patterns, such as '*X gene*' and '*the expression of X*'. The context patterns were extracted from BioCreative 2 corpus and ranked by information gain, and then the top ranking entries were selected as CDFs. We denoted a gene name candidate mentioned in a sentence by the token sequence



where the *NE *refers to the entity, *t*_-*n *_is its previous *n*th token and *t*_+*n *_is the next *n*th token. The detailed process is as follows: 1) use the dictionary to search the BioCreative 2 training corpus and get the set of *ts*, such that *NE *is in the dictionary for each *ts*. 2) For each *ts *build a feature vector from the context patterns



where each pattern is treated as a binary feature. 3) Assign an example positive if it is at the answer position (gold or alternative standard) of the BioCreative 2 training data, and negative otherwise. 4) Rank features using information gain and select top 300 left and 300 right contextual patterns as CDFs. Some examples of these features are listed in Table 2. In addition, we found the features derived from term frequencies of EDFs could be used to enhance the classification performance, since gene names tend to low-frequency than noisy terms. Interestingly they can also be attributed to FCD features, where the CDF can be viewed as the deliminator of words. We attribute it to CDF I.

#### CDF II

outputs of another classifier. There are two problems in CDF I: 1) one pattern only considers one term in context, which limits its distinguishing ability. 2) Since only top ranking ones are considered, there may exist a certain amount of highly indicative contexts that cannot be utilized. Addressing these problems, we used the outputs of a classifier trained by local contexts as another type of CDF, so that the two types could be complementary. This classifier was to predict the class label of the current name candidate by its surrounding contexts. We used the examples generated in the previous step of selecting CDF I and converted local context words in a window [-5, +5] into features for each example. Features were the conjunction of context words and the offsets from the *NE *(e.g., '*word = human∧offset = -1*'), and the token numbers of the name candidate. SVM^light ^with linear kernel were used, where the parameter *C *was set at 0.1. Since the outputs of SVM were real numbers but the CDFs we considered were binary features, the SVM scores were discretized into several intervals, and each interval was treated as a CDF. Table 2 lists all the features of this type.

### FCD measures

First we analyze the original PMI [19] and the PMI variant used in *KnowItAll *[13]:

(3)

(4)

where *co(x, y) *is the co-occurrence count of *x *and *y *in the corpus and *N *is the total word count. According to our framework, *x *is an EDF, and *y *is a CDF. As discussed in the previous sections, we argue that the FCD metrics should be selected according to the base classifier and the target problem.

For example, as suggested by PAC-Bayes margin bound [20], margin based classifiers, such as SVM, prefer 'normalized' features, where the norm of input feature vector has played an important role in the generalization error bound. In addition, the relative diameter of the input data is a key factor of generalization error bound for a variety of classifiers [21]. Apparently the PMI in formula (3) is not normalized well. Also both in formula (3) and formula (4), the relative diameters of the input data are large due to the high discrepancies between term frequencies in large data corpus. Thus the score will be dominated by high frequency terms. This bias will be more apparent in formula (4) because there is no logarithm to scale the count as formula (3). And the impact of CDF count is not considered in formula (4). Considering these factors, we propose another FCD metric:

(5)

where the term count is replaced by its logarithm value, and b was assigned 1 in our experiment. In this simple way, data becomes more centralized and normalized. We also investigate a simple FCD metric:

(6)

In this formula only the co-occurrence count and the total word count are considered, which can be viewed as a scaled joint probability distribution of two indicative features. It emphasizes the impact of co-occurrence count more than formula (5) and we expect it can be used as a complementary measure for *FCD1*. If the CDF is very weak and the *co(x, y) *are very close to *count(x)*, the impact of the co-occurrence count in formula (6) can be ignored, but we do need this value in some cases. For example, if the CDF is the word deliminator as mentioned above, *FCD1 *will not be suitable but *FCD2 *can be used instead. In our experiment, both *FCD1 *and *FCD2 *are used for features related to CDF II, and only *FCD1 *are used for CDF I (left and right types). Because the vocabulary size of CDF I is much larger, using both metrics will double the feature space. In addition, we will compare the single performance of formula (5) and formula (6) in order to investigate what factors are most important in these PMI-style FCD metrics.

### Unlabeled data

The scale of unlabeled data is an important factor to ensure that sufficient co-occurrences can be obtained to prevent the data sparseness in FCD features. Although the explosion of biomedical literature brings a big challenge for biologists, it has provided rich resources for semi-supervised learning, e.g., MEDLINE records. Our data collection was derived from two sources: 1) MEDLINE records (English) up to Nov. 2007 (totally 13,781,184 articles). The title and abstract fields were used in this work. 2) Data collection of TREC 2006 Genomics track [22], which consists of 162,259 full text articles from 49 journals. The preprocessing method was the same as our prior work [23], where non-narrative texts such as titles, affiliations, tables, and references were removed, and special characters were converted to corresponding strings in MEDLINE (e.g., α-> alpha). Using these data we were able to investigate the relationship between the unlabeled data scale and the classification performance. To get the term frequencies and co-occurrence counts in the huge text corpus, we directly searched the text and got the required information at one time for all the dictionary entries, rather than built an inverted index as Web search engines.

### Classification model

We used SVM as the classifier because it performs well in high dimensional feature space. In addition, since it is a kernel based method, it is convenient to investigate the nonlinear characteristic of FCD features using nonlinear kernels. Different from lexical features, the dimension of FCD features is much smaller and the density of feature space (around 25% in our experiments) is much higher. Interestingly we found that this feature space was somewhat like that in the task of *image recognition*. Inspired by the prevailing techniques in such tasks, we first used singular value decomposition (SVD) to get a subspace of the original features and then used a SVM with a radial basis function (RBF) kernel to classify the examples. We performed SVD to the training examples and selected the most significant *k *columns of the left singular matrix as new training examples, and at predicting time, examples were generated by inner product computation. The parameter *k *of SVD and γ of RBF kernel (by the -g option in SVM^light^) were both set at 300 in the final runs.

In addition, in order to combine the lexical features and FCD features we integrated all the features in a linear kernel. However, since the two views were heterogeneous we wondered whether they could co-operate well in the same regularization framework. Also the nonlinear information of FCD features could not be incorporated in this way. Therefore we used a simple ensemble method to combine the outputs of the linear model (lexical and FCD features) and the SVD-RBF model (FCD features only). They were integrated in a weighted linear function, where the weights were assigned 2/5 and 3/5 respectively, and the decision threshold was -0.2.

### Named entity recognition

The NEC system (Figure 4) assigned each dictionary entry a confidence score that told how likely it was a gene name. Based on this information, we developed two simple methods for NER: a dictionary look-up and a combined method. The dictionary look-up was a simple forward maximum match, which considered only dictionary entries with decision scores higher than -0.2. Entries in the dictionary were organized in a prefix Trie tree. The tagging process was rather efficient (around 10,000 sentences per second).

The combined method consisted of two steps: first, we used the results of the dictionary look-up as features for a CRF model. Then we combined the outputs of CRF and high confident entries in the dictionary (decision score higher than 0.2). For overlapping entries we chose the results of dictionary look-up.

Our CRF-based tagger was a modified version of ABNER system [7,24], an open source tool for biomedical named entity recognition. We did three simple modifications in its code: 1) case-sensitive word features were added. 2) The window size of feature conjunction was augmented to [-2, -1, 1] from [-1, 1] and all the features were united by this methods. 3) We used labels denoted by I-E-O (inside-end-outside), which yielded a slightly better performance than B-I-O (beginning-inside-outside) in our experiment.

Two types of dictionary features were used here:

*Strict match features*: conjunction of 'is in dictionary' and token number of the dictionary entry: for example, if term '*murine beta gene' *is found in the dictionary, each word will be assigned the feature '*IsInDic∧Len = 3*'.

*Prefix match features*: conjunction of 'part in dictionary' and 'depth of prefix'. For example, if the term '*murine beta' *is the prefix of a dictionary entry, the two tokens will be assigned the feature '*PrefixDepth = 1*' and '*PrefixDepth = 2*' respectively. It is a fuzzy match method and is expected to improve the recall of dictionary features.

Since the data structure of our dictionary is a prefix tree, these two features can be extracted at one time. This means the extraction of 'prefix match features' almost does not need additional computational cost. Note that for dictionary features the overlap matches were allowed rather than maximum match used in the dictionary-based recognition. In addition, we found that degrading the weights of dictionary features lead to a slight improvement on the final performance. Here we set the weight of dictionary features at 0.1 and the other features at 1.0. The data/process flow diagram of the NER system is shown in Figure 1.

## Results and discussion

### Lexical features vs. FCD features

Table 3 shows the performance of lexical features and contributions of each feature type in the named entity classification task. It can be seen that each feature type improves the classification performance significantly and the best F-score achieved by lexical features is 80.26. We also examine the performance on OOV terms, which are defined as name candidates in which any word is not in the training data, such as '*exuperantia*', '*bacteriorhodopsin*', '*serine49*', '*phycoerythrin hexamers*', '*m13mp19*' and '*mihck*' in our experiment. The proportion of OOV terms in the test set is around 10%. Table 3 shows that the performances on OOV terms are much inferior. The n-gram based features (F1 and F2) are not able to represent OOV terms defined here. The substring based features (F3 and F4) and morphology patterns (F5) make the OOV term classification feasible, but the F-score is much lower than the overall performance. It indicates that the biased representation of old and new names is one serious drawback of lexical features, which has proved our analysis in the introduction section.

**Table 3:** Performance of lexical features on named entity classification. F1: bag-of-n-grams; F2: boundary n-grams; F3: sliding character window; F4: boundary substrings; F5: morphology patterns.

Feature	All terms (P/R/F1)	OOV terms (P/R/F1)
F1	53.97/70.72/61.22	--
F1+F2	73.98/67.13/70.39	--
F1+F2+F3	72.54/71.15/71.84	50.88/2.94/5.56
F1+F2+F3+F4	73.75/85.22/79.07	70.93/75.89/73.32
F1+F2+F3+F5	72.81/**86.46**/79.05	71.00/74.16/72.55
F1+F2+F3+F4+F5	**75.52**/85.63/**80.26**	**74.03/75.68/74.85**

From Table 4, it is surprising to find that all the models with FCD features significantly outperform those with the lexical features only. In particular, the improvements on OOV term classification are over 10 points in F-score. The SVD-RBF model (Run 3) has both higher precision and recall than the linear case on overall performance. In Run 4 we used a simple weighted linear function to combine the outputs of Run 2 and Run 3, where the weights were set at 1/4 and 3/4 respectively. Note that it is the best run produced by FCD features only. Interestingly, it outperforms the classical methods by 5.97 F-score and is *greatly different *from all the previous methods used in biological NER/C tasks, since not any lexical features or domain specific regular expressions are used here.

**Table 4:** Performance of models with FCD features on named entity classification and recognition

ID	Feature(model)	Classification (all terms)			Classification (OOV terms)			Named entity recognition		
		Precision	Recall	F-score	Precision	Recall	F-score	Precision	Recall	F-score
Run 1	Lexical (linear)	75.52	85.63	80.26	74.03	75.68	74.85	85.70	78.36	81.86
Run 2	FCD (linear)	81.59	87.77	84.57 (+4.31)	83.74	87.64	85.64 (+10.79)	87.98	80.70	84.18 (+2.32)
Run 3	FCD (SVD + RBF)	83.02	88.24	85.55 (+5.29)	83.12	85.31	84.2 (+9.35)	89.80	81.76	85.59 (+3.73)
Run 4	FCD (Combine (2, 3))	82.46	**90.35**	86.23 (+5.97)	83.21	88.35	85.7 (+10.85)	89.29	**82.45**	85.74 (+3.88)
Run 5	All (linear)	82.96	89.31	86.02 (+5.76)	83.65	**89.16**	86.32 (+11.47)	89.93	81.71	85.62 (+3.76)
Run 6	All (Combine (3, 5))	**83.94**	89.99	**86.86 (+6.6)**	**83.92**	88.86	**86.32 (+11.47)**	**90.37**	82.40	**86.20 (+4.34**)

In Run 1, 2 and 5 SVMs with linear kernel are used. In Run 3, SVD is used to reduce the feature dimension and a SVM with RBF kernel is used to classify examples. In Run 3 only features related to CDF I are used. In Run 4 outputs of Run 2 and 3 are combined. Run 6 is the combination of Run 3 and Run 5.

The performance discrepancy between Run 4 and Run 6 reflects the additional contribution of lexical features based on the best setting of FCD features, where the improvements are merely around 0.6 in the two kinds of F-score. It seems that the lexical features are *replaced *by the FCD features and become somewhat *redundant*. In other words, the FCG method has transformed the sparse lexical features into a lower dimensional and more informative feature representation while little information is lost.

In Table 4, a slight improvement on the NEC task yields positive impact on the NER task, indicating that the NEC task can be especially valuable for NER. But the improvement on NER is relatively smaller than that on NEC, because the NER task is evaluated in a plain text corpus, where the performance of high frequency terms has much bigger impact on the F-score, but the advantage of FCD features focus on identifying OOV terms which tend to be less frequent in the test corpus. Detailed discussion about NER is presented in the 'Named entity recognition' sub-section.

### Impact of CDFs

In Table 5 performances of different CDFs are compared. In all the runs both EDF I and EDF II were used. CDF I performs much better than CDF II, which indicates that we can get rich information to identify a gene name by integrating large amount of term co-occurrences in global context even though one CDF in a local context is not strongly indicative. The introduction of CDF II leads to a further improvement over CDF I and the space cost is rather low since the dimensionality of CDF II is much smaller. Therefore, based on this type of feature we can develop many other types of EDFs, combine several FCD metrics or incorporate more features into the classifier in the generation procedure of CDF II. In our experiment, we found that when combined with other linear models, the RBF model with CDF I outperformed that with CDF I+II slightly but consistently though its single performance was inferior. One explanation is that for ensemble learning it is important to make the sub-classifiers different. The former model was used in all the combining models in our experiments.

**Table 5:** Impact of different CDFs on named entity classification.

CDF type	Linear (P/R/F1)	SVD-RBF (P/R/F1)
CDF I	81.43/86.50/83.89	**83.02**/88.24/85.55
CDF II	75.64/82.20/78.78	78.41/80.35/79.37
CDF I+II	81.59/87.67/84.57	82.51/90.01/86.10
Combine	82.46/**90.35**/**86.23**	--

The run in the last row combines the results of SVD-RBF model (with CDF I) and linear model (with CDF I+II), which is the same as Run 4 in Table 4. Since the combining method is a linear function, we attribute it to the linear case.

**Figure 5:**
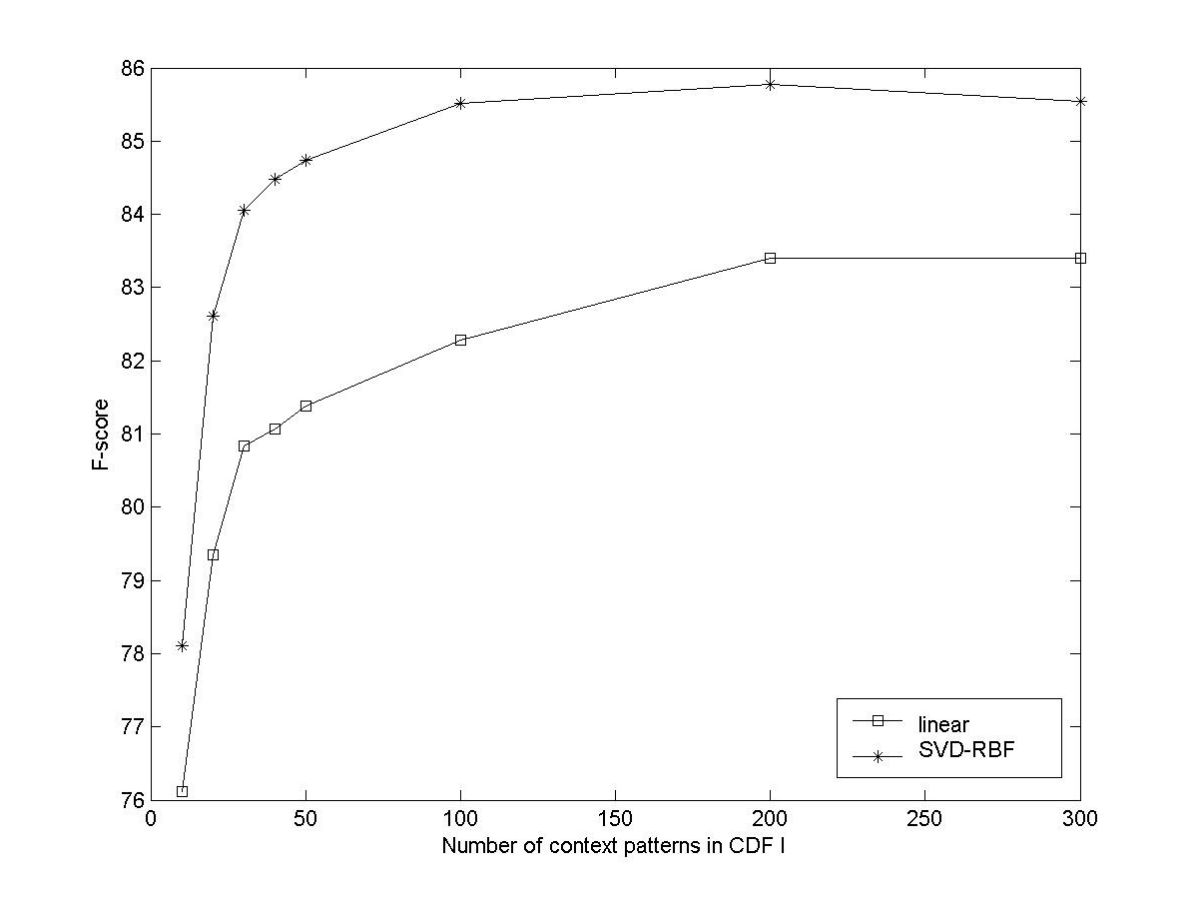
**Relation between named entity classification performance and the number of context patterns in CDF I**. The patterns are selected in a descendent order of information gain scores.

Figure 5 shows the impact of indicative patterns of CDF I on the classification performance. It is interesting to see that only a small amount of terms lead to better performances than lexical features. In addition, with the CDF number increasing the F-score improves steadily for linear SVM. The overall performances of the SVD-RBF model are better than those of the linear model, but when the number of terms reaches certain amount (around 200) the performance of nonlinear model decreases slightly. We think it is mainly because SVD is an unsupervised method, where features of different classes may be merged when seeing a lot of irrelevant features and important information can be lost. Previous Web-based methods [8,13] only considered a small amount of context patterns, but in our experiment, we have proved that the performance can be substantially enhanced by incorporating much more indicative context patterns.

### Impact of EDFs and unlabeled data

Table 6 shows the performance of different EDFs. In these runs, both CDF I and CDF II were used, and the classifier was a linear SVM. It can be seen that the introduction of EDF II improves the classification performance by 6.2 F-score. Since there are a large number of multi-token entity names in biomedical literature and many exact co-occurrences of these names and CDFs are difficult to obtain from the unlabeled data, the performance of EDF I alone is only 78.37. Features of EDF II are boundary n-grams, which are less indicative but more informative than EDF I. This means we can get more feature co-occurrences from unlabeled data. It indicates that if sufficient information is not available from unlabeled data, 'softening' the EDF by selecting less discriminative but more informative features is an effective way.

**Table 6:** Performance of different EDFs on named entity classification

CDF type	Precision	Recall	F-score
EDF I	77.07	79.71	78.37
EDF I + EDF II (1-gram)	80.73	86.90	83.70 (+5.33)
EDF I + EDF II (1,2-gram)	81.17	87.57	84.24 (+5.87)
EDF I + EDF II (1,2,3-gram)	**81.59**	**87.77**	**84.57 (+6.2)**

**Figure 6:**
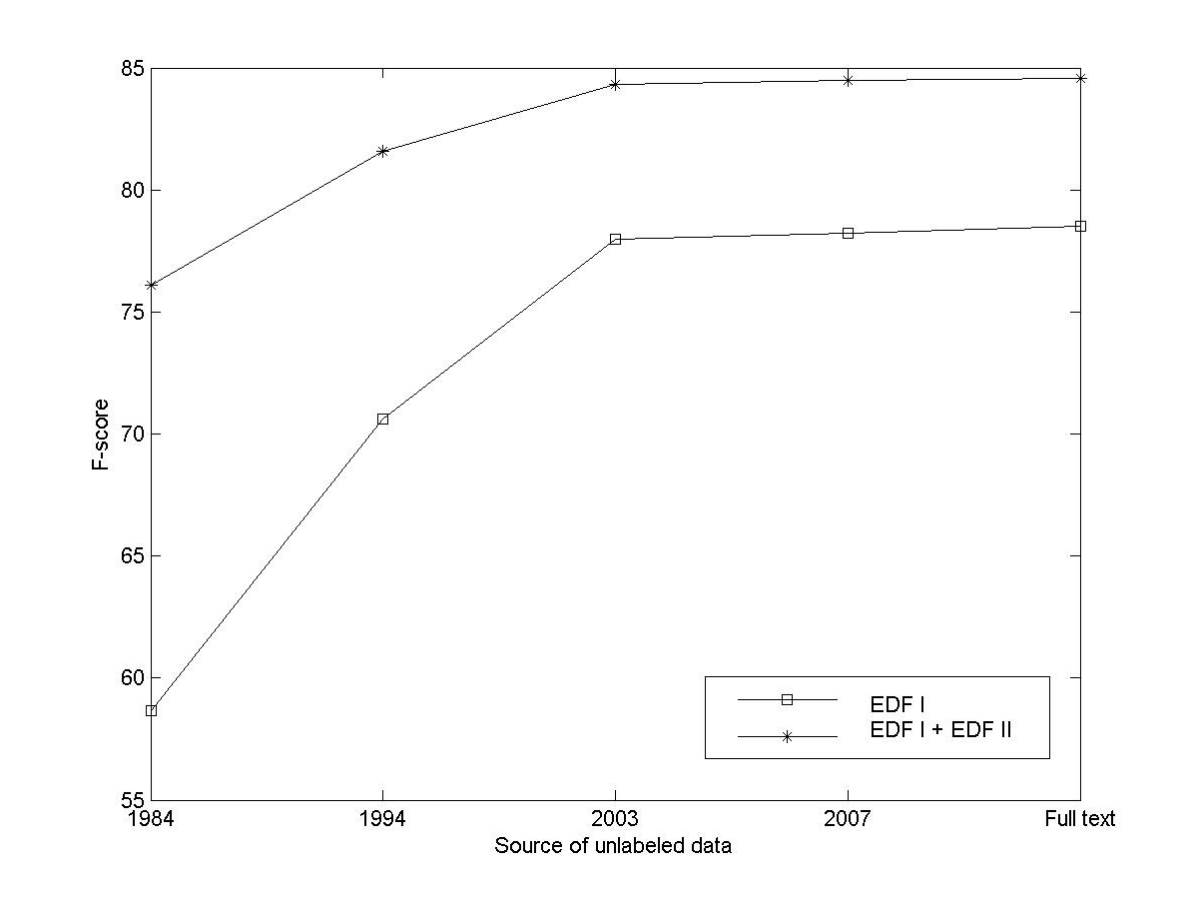
**Relation between named entity classification performance and unlabeled data**. The years are the final publication years of MEDLINE abstracts. The 'Full text' includes all the MEDLINE abstracts and TREC 2006 Genomics Track data collection.

Figure 6 describes the impact of unlabeled data on the classification performance. It can be seen that the advantage of EDF II is even more apparent when the size of unlabeled data is relatively small. Also it is promising to see that the performances always improve when more unlabeled data is added. The cheap unlabeled data has become a valuable resource of background knowledge. However the additional improvement caused by unlabeled data becomes very slight after adding the ten-year-MEDLINE (1994–2003). It seems that the unlabeled data has already been exploited fully and significant gain cannot be expected by augment of unlabeled data simply. Our method is able to utilize huge amount of unlabeled data easily, which scalability outperforms all the current semi-supervised methods presented in the survey [14].

### FCD measures

In Table 7 several FCD metrics are compared, including methods used in the previous works [8,13] and formulas presented in previous sections. For simplicity we only consider the results of linear SVM. As can be seen, the performance of binary features is much lower since it ignores the degree of co-occurrence. *PMI*_*KnowItAll *_considers both co-occurrence and EDF counts, achieving 1 point higher F-score than the binary case. The original PMI and normalized PMI outperform *PMI*_*KnowItAll *_by over 1 point. We think the main reason is the use of logarithm to centralize the data. Normalization of original PMI leads to improvements in both precision and recall, but for other metrics the effects are very little (not reported in this paper), as the norms of input vectors are already small in these metrics.

**Table 7:** Comparison of different FCD metrics

FCD metric	Precision	Recall	F-score
Binary	77.60	82.39	79.92
*PMI*_*KnowItAll*_	78.74	83.22	80.92 (+1.00)
PMI	79.51	84.84	82.09 (+2.17)
Normalized PMI	79.83	85.33	82.49 (+2.57)
*FCD2*	81.42	87.35	84.28 (+4.36)
*FCD1*	81.52	87.54	84.42 (+4.5)
*FCD1*+*FCD2*	**81.59**	**87.77**	**84.57 (+4.65)**

In addition, as can be seen from the last three rows of Table 7, significant improvements are achieved when the logarithm version co-occurrence count is used. Logarithm sublinear scaling is a commonly used technique in IR domain. For example, log term frequency (TF) is one of the most effective methods of term weighting schemes [25]. According to the statistical learning theory [21], this can also be interpreted by the centralization of the data. Using this method the radius of the data *R *becomes much smaller but the margin *Δ *does not shrink much, so the generation error bound determined by *R*^2^/*Δ*^2 ^becomes smaller. Another conclusion is that the co-occurrence count is the most important factor in these FCD metrics, since there are tiny differences between runs with *FCD1 *and *FCD2*.

### Named entity recognition

In Table 8 and Table 6, it can be seen that the simple dictionary look-up method is able to achieve an 86.2 F-score, an improvement of 4.34 points over the dictionary derived from our lexical features. It is much higher than the other dictionary based methods used in the challenges [2-4], and only 1.01 points lower than the best F-score in BioCreative 2 challenge [3]. Run 3 (Table 6) does not use *any previous classical features *and is able to achieve an F-score of 85.74. There are two main advantages of dictionary based recognition methods. First, it is more efficient than linear discriminative model with sequential decoding. Second, it makes gene name normalization easier, since it directly maps the gene identifiers in database. Most systems in BioCreative 2 gene normalization or protein-protein interaction (PPI) tasks used dictionary-based NER as initial steps.

**Table 8:** Comparison of performance and applicability of different NER systems on BioCreative 2 test set

System or authors	Precision	Recall	F-score	# of features	Tagging complexity	Availability
CRF 1 (ABNER+)	87.30	80.68	83.86	171,251	LM	N
CRF 2 (ABNER++)	87.39	81.96	84.59	355,461	LM	N
Dictionary	90.37	82.40	86.20	**0**	**Trie**	**Y**
Dictionary + CRF 2	**90.52**	87.63	**89.05**	355,609	LM	**Y**
BANNER [26,27]	88.66	84.32	86.43	500,876	LM+POS tagger	**Y**
Ando [5] (1^st ^in BioCreative 2)	88.48	85.97	87.21	--	2*LM+POS tagger+syntactic parser	N
Hus *et al*. [10]	88.95	**87.65**	88.30	8 * 5,059,368	8*LM+POS tagger	N

In the 6^th ^column, 'LM' and 'Trie' respectively refer to the time complexities of a linear model and a Trie tree based dictionary match. The 'Dictionary' method doesn't need any feature, once the dictionary is constructed. For Ando's system, we cannot find the number of features in the paper [5]. Since the systems in the last two rows used classifier combination, the tagging complexities and numbers of features are multiplied by the numbers of sub-models.

In the first run in Table 8, we simply compiled the source code of ABNER to train the tagger based on the BioCreative 2 training data. The second run denoted by ABNER++ is the model with modified feature set using the three tricks as described previously, which results in an improvement of 0.73 points. In particular, it is encouraging to see that the 4th run that combines the dictionary and CRF model using simple methods (given in the previous section) has achieved an F-score of 89.05, which outperforms all the previous systems on the same dataset.

Note that in all the runs we do not use POS tagger, syntactic parser, domain specific post-processing or multiple classifier combination [5,8,10], as our focus in this paper is to investigate the gain from background knowledge and FCG method, and we also consider the tagging efficiency. Of course, combining our dictionary with more elaborately designed features and methods may produce higher results.

The tagging speed is a very important factor for a successful text mining tool in the presence of huge amount of biomedical literature. Addressing various criteria for evaluating NER systems in practice, we compare the performances, feature sizes, tagging complexities, and availabilities of current state-of-the-art gene NER systems, including an open source software BANNER [26,27], which is also trained by the BioCreative 2 training data and yields 86.43 F-score (the latest version) on the test set. The available service of AIIA-GMT [10] is related to the work [10], but the tagger was trained by the test corpus of BioCreative 2, so we cannot compare it with our systems.

For sequence labeling, the run time complexity of a linear model can be approximately estimated by O(Te*N +Se*N), where *N *is the average length of a sequence (usually a sentence in NER), *Te *is the number of feature templates (or feature generators) and *Se *is the average search length for a feature. For simple features, the impact of *Te *and *Se *can usually be ignored, since template match and Hash search can be very fast. However in the model with millions of lexical features, especially when using shallow or syntactic parsers, the time for generating and accessing features will become dominative. For a CRF model, the number of states, the order of Markov chain and Viterbi decoding will also add additional time cost. For Trie-based dictionary match, the run time complexity is O(Le*N), where *Le *is the average search length for a dictionary entry, which is less than the height of the Trie tree (the maximum length of dictionary entries). On the 5,000 testing sentences of BioCreative 2 GM task, the dictionary look-up and CRF model cost around 0.5 s and 15 s respectively. Thus we ignored the impact of dictionary look-up in the combined method (Table 8). It can be seen from Table 8 that our systems ('Dictionary' and 'Dictionary + CRF 2') are more efficient than other available systems with similar performances. The reason is that we transfer the computational cost into the dictionary construction, which can be done in an offline manner.

## Conclusion

We presented a general framework for gene named entity representation and showed its contribution to the NEC and NER tasks. The experimental results show that it is able to learn a higher level representation, which leads to better classification/recognition performances than elaborately designed lexical features that are widely used in previous researches. We believe that there is still large room for this method to be further improved. For example, better EDFs, CDFs, FCD types or unlabeled data can be considered. Also since the characteristic of FCD features are very different from sparse lexical features widely used in text mining, more classical pattern recognition methods can be examined, such as kernel or PCA. In addition, it can be easily incorporated into other supervised or semi-supervised methods, since our method just results in new features.

FCG is capable of utilizing huge amount of unlabeled data and simple to implement, which is the advantage over other semi-supervised learning methods [14]. However, it needs to visit unlabeled data to get co-occurrence counts at predicting time, which may need the help of efficient distributed search engines for online applications. Methods based on commercial Web search engines (e.g., Google) would be limited by traffic, availability, down times or domain adaptations, etc. Also many EDFs and CDFs (e.g., CDF II) cannot be obtained, since the index units of Web search engines are only words or phrases rather than general features. One solution is to build a 'feature-level' search engine for specific tasks. Another simple way as we did in our experiments is to collect all the statistics by reading the unlabeled data in an offline manner. This requires the application can be divided into 'non-real-time' subtasks. For example, our NEC task is such subtask of NER, where once the dictionary is built, the recognition speed can be very fast, though the construction of dictionary is a time-consuming work. Interestingly it is somewhat like the procedure of human learning.

Although the method is inspired by NEC and NER, the idea of FCG is of independent interests. It will be very interesting to examine this method in other applications, since it is general enough to solve many sparseness problems given a large amount of unlabeled data. Also quantification of the factors that influence the EDF or CDF generation can be especially useful for general purpose.

Our two-stage method for NER provides an effective way to incorporate external dictionary resources, because if we first remove the 'noise' in the dictionary, its NER performance will be enhanced significantly. The experimental results show that in the NER task an automatically constructed dictionary can bring around 5% F-score improvement over rich lexical feature based CRF model, which is significantly higher than the results in previous studies. In addition, our dictionary contains confidence scores on how likely an entry is a gene or protein name in most cases, which makes the trade-off between precision and recall of NER can be easily tuned. Besides NER, it can also be used for query expansion in IR tasks, gene named entity normalization or protein-protein interaction.

## Authors' contributions

YL proposed the methods, designed the experiments, implemented the systems and drafted the manuscript. HL and ZY guided the whole work and helped to revise the manuscript. All authors have read and approved the final manuscript.
